# Distinct gender differences in anthropometric profiles of a peri-urban South African HIV population: a cross sectional study

**DOI:** 10.1186/s12879-015-0836-9

**Published:** 2015-02-21

**Authors:** Theodore A Nell, Maritza J Kruger, Dillan C Beukes, Esme Calitz, Rehana Essop, M Faadiel Essop

**Affiliations:** Cardio-Metabolic Research Group (CMRG), Department of Physiological Sciences, Stellenbosch University, Mike De Vries Building, Merriman Avenue, Stellenbosch, 7600 South Africa; Division of Community Health, Tygerberg Campus, Stellenbosch, 7600 South Africa; TC Newman Community Day Care Centre, Paarl, 7646 South Africa; ANOVA Health Institute, Kohler Street, Paarl, 7646 South Africa

**Keywords:** HIV, Anthropometry, Body composition, HAART, Peri-urban, South Africa

## Abstract

**Background:**

Highly active antiretroviral therapy (HAART) has extended life expectancy and enhanced the well-being of HIV-positive individuals. Since there are concerns regarding HAART-mediated onset of cardio-metabolic diseases in the long-term, we evaluated the anthropometric profile of black HIV-infected individuals in a peri-urban setting (Western Cape, South Africa).

**Methods:**

A cross sectional study design was followed to describe the gender differences in different HAART treatment groups. HIV-positive patients (n = 44 males, n = 102 females; 20–40 years) were recruited for three groups: 1) control (HIV-positive, HAART-naïve), 2) HIV-positive (<3 years HAART), and 3) HIV-positive (>3 years HAART).

**Results:**

All participants underwent comprehensive anthropometric and bio-electrical impedance analyses. No significant differences were observed in the male treatment groups. HAART-naïve females are mostly overweight (73.90 ± 2.79). This is followed by a period of muscle wasting seen in the triceps skinfold (29.30 ± 2.19 vs 20.63 ± 1.83; p < 0.01), muscle mass (22.23 ± 0.46 vs 19.82 ± 0.54; p < 0.01), and fat free mass (49.40 ± 1.08 vs 44.16 ± 1.21; p < 0.01) upon HAART initiation (<3 years HAART). Thereafter all parameters measured had levels similar to that seen for the female HAART-naïve group. Females on <3 years HAART exhibited significantly decreased body cell mass (p < 0.01), protein mass (p < 0.01), muscle mass (p < 0.01), fat free mass (p < 0.01), and fat mass (p < 0.001) versus matched HAART-naïve controls. The W:H ratio for the female treatment groups placed the females overall at a higher risk for developing cardiovascular disease compared to the males.

**Conclusions:**

This study found striking gender-based anthropometric differences in black South African HIV-positive individuals on HAART. We also conclude from this observational study that no significant differences were found in the different male treatment groups. All female body composition parameters initially showed lower values (<3 years HAART). The female treatment group (>3 years HAART) displayed values similar to that seen in the HAART-naïve group. Higher W:H ratios in females receiving longer-term HAART potentially increases their risk for the future onset of cardio-metabolic complications.

## Background

South Africa is currently burdened with the highest global prevalence of HIV-AIDS, with ~6.4 million HIV-infected individuals making up ~12.2% of the population [1]. Although the increased roll-out of highly active antiretroviral treatment (HAART) is blunting the spread of HIV-AIDS and extending life expectancy [2], there are growing concerns regarding longer-term co-morbidities such as the onset of type 2 diabetes and cardiovascular diseases (CVD) [3-6]. In parallel, recent studies demonstrate that increased obesity, and especially abdominal fat, is associated with a greater risk for CVD onset within the wider populace [7,8]. Since the broader South African population is also faced with an increasing obesity prevalence, the country is strongly challenged by a so-called dual burden of disease [2]. This burgeoning health issue will result in serious consequences by impacting on the overall well-being and health of its population, and also by stunting economic growth and development.

Previous studies show that HAART can trigger body composition changes leading to visceral fat accumulation in HIV-infected persons [9]. Moreover, ~30% of HAART-treated individuals display some evidence of anthropometric and body composition changes, including weight gain, fat redistribution and other significant metabolic alterations [10,11]. Despite such concerns, limited research has been conducted on sub-Saharan African populations to evaluate potential cardio-metabolic risk in HIV-positive persons. Here the emphasis has largely been placed on evaluating cardio-metabolic co-morbidities in developed countries [3]. However, a recent study performed on HIV-positive South African urban women found that HAART was associated with increased central fat gain and decreased levels of peripheral fat [12]. In agreement, others reported that HIV-positive individuals exhibited subcutaneous fat displacement accompanied by muscle wasting [13].

In light of this paucity of data within the South African HIV-infected population, the current study assessed anthropometric changes within a peri-urban setting (Paarl, Western Cape, South Africa). We also set out to provide novel insights into the question of gender-based anthropometric changes in response to HIV-positive persons on HAART since this remains relatively poorly understood.

## Methods

### Design and subjects

This cross-sectional study was performed during 2012 and 2013 and evaluated gender-based body composition differences between HIV-positive HAART and HAART-naïve individuals. Black South African males and females (n = 146) were randomly recruited and selected from ~1, 500 out-patients at the TC Newman Community Day Care Centre and the Mbekweni Clinic from the Paarl and Wellington health districts, respectively. Paarl and Wellington are closely located neighboring towns within a peri-urban setting, and located ~35 miles from Cape Town (Western Cape, South Africa). HIV positive males or females that are on treatment, and/or newly diagnosed HIV patients (for controls); age ranges between 20 and 40 years; residents of Paarl, Wellington and the Drakenstein region were included in the study. No pregnant or lactating females or patients diagnosed with acute diseases were entered into the study. Participants were subsequently divided into two arms i.e. a) HAART-naïve control group (n = 48 [32.9%]; n = 13 males [8.9%] and n = 35 females [24.0%]), and b) HIV-positive HAART groups (n = 98 [67.1%]; n = 31 males [21.2%] and n = 67 females [45.9%]), respectively. The HAART group was further split into two sub-groupings, i.e. HAART for less and longer than three years, respectively. Such sub-groups were subsequently analyzed in a gender-dependent manner. We employed this particular sub-group classification based on previous research that found that HAART-induced anthropometric and metabolic alterations manifested in a temporal manner [14].

### Ethical considerations

Both the Human Research Ethics Committee of Stellenbosch University and the Western Cape Government Ethics Committee approved this study (S12/03/073 and 2012 RP 100). Written consent was obtained from all participants after volunteering and agreeing to take part in the study. Participants were also free to withdraw from the study if they no longer wished to continue and had access to their standard medical care throughout the duration of the study.

### Testing procedures

All participants were informed of the physical testing procedures. Conventional anthropometry was performed in duplicate and included base measurements, circumferences and skin folds. Height was measured to the nearest 0.1 cm with a portable stadiometer (Seca United Kingdom, Birmingham, England), while weights were determined (with minimal clothing) to the nearest 0.01 kg using a calibrated Seca 634 scale (Seca United Kingdom, Birmingham, England). Waist circumference (WC) was measured with a Lufkin® executive thin line tape measure (Lufkin W606PM) (Apex Tool Group, USA). The circumference was determined at the narrowest point between the lower costal border and the top of the iliac crest perpendicular to the long axis of the trunk [15]. The hip circumference (HC) was taken at the greatest posterior protuberance of the gluteal muscles, while the body mass index (BMI; kg/m^2^) and waist-to-hip (W:H) ratio were calculated by employing the parameters here described. The triceps skinfold (TSF) was measured using a skinfold caliper (Harpenden, Baty International, West Sussex, UK), and the TSF site was located at the point on the posterior area of the right arm in the mid-line of the level of the mid-acromiale radiale landmark.

Bio-electrical impedance analysis (BIA) was completed by employing a Maltron Bioscan 9200 standard tetra-polar multi-frequency analyzer (Maltron International, Rayleigh, Essex, UK) that analyzes the percentage fat, fat mass, body cell and muscle mass. Here a BIA pre-test protocol was implemented, i.e. a) emptying of the bladder, and b) removal of all metal objects from the skin. In addition, participants were instructed to lie on the examination bed with their legs and arms slightly apart with no limbs touching the body, while conduction electrodes were placed on both the right hand and right foot.

### Statistical analyses

Statistical analyses were performed using Statistica version 12 software (Statsoft, Tulsa OK, USA). The means, standard deviations and standard error of the mean (SEM) were calculated for all parameters, and factorial analysis of variance (ANOVA) employed to evaluate differences between various groupings and sub-groupings here investigated. The Bonferroni *post-hoc* test was subsequently used to assess the significance of differences found between groups. We also completed additional analyses with the W:H data (as marker of fat distribution), i.e. Spearman correlation co-efficient was performed on W:H and CD4 cell counts. *P*-values of ≤ 0.05 were considered statistically significant and the mean ± SEM was used for all results reported.

## Results

### Participant and body composition characteristics

Of the 146 participants included, most were female (n = 102; 70%) with a mean patient age of 30.9 ± 0.58 years, compared to 30% males (n = 44) with a mean patient age of 33.3 ± 0.69 years (refer to Tables 1 and 2 for summary of descriptive characteristics). We initially assessed the data in a gender-dependent manner for all of the study participants (HIV-positive HAART and HAART-naïve). Here our data reveal that female participants displayed increased levels of adiposity as determined by anthropometric analysis. These data were confirmed by the BIA analysis that showed lower levels of fat free mass in female participants. In addition, females exhibited attenuated levels of glycogen and total body potassium and calcium (Table 2).

**Table 1:** **Summary of anthropometric characteristics for all participants, irrespective of group specifics**

**Variable**	**Male**	**Female**	***p*** **-value**
	**(n = 44)**	**(n = 102)**	
**Age (years)**	33.30 ± 0.69	30.90 ± 0.58	p < 0.05
**Height (cm)**	170.80 ± 0.84	157.90 ± 0.63	p < 0.001
**Weight (kg)**	62.70 ± 1.60	70.80 ± 1.80	p < 0.01
**BMI (kg/m** ^**2**^ **)**	21.50 ± 0.54	28.30 ± 0.70	p < 0.001
**Triceps skinfold (mm)**	9.60 ± 1.10	27.50 ± 1.30	p < 0.001
**Mid-upper arm circumference (cm)**	26.70 ± 0.55	30.90 ± 0.60	p < 0.001
**Waist circumference (cm)**	76.80 ± 1.60	90.60 ± 1.50	p < 0.001
**Hip circumference (cm)**	89.30 ± 1.20	105.30 ± 1.40	p < 0.001
**Waist-to-height ratio**	0.45 ± 0.01	0.57 ± 0.01	p < 0.001
**Waist-to-hip ratio**	0.86 ± 0.01	0.86 ± 0.01	-

Values represent mean ± SEM. All male subjects were compared to female subjects according to their anthropometric characteristics. Student t-tests were used and p<0.05 considered as statistically significant.

**Table 2:** **Summary of BIA characteristics for all participants, irrespective of group specifics**

**Variable**	**Male**	**Female**	***p*** **-value**
	**(n = 44)**	**(n = 102)**	
**CD4**	314.69 ± 39.92	388.51 ± 23.49	-
**Body cell mass: Fat free mass**	0.58 ± 0.002	0.55 ± 0.00	p < 0.001
**Resting metabolic rate (kcal)**	1776.41 ± 20.64	1553.73 ± 10.55	p < 0.001
**Fat free mass (kg)**	53.46 ± 0.95	48.29 ± 0.70	p < 0.001
**Fat free mass (%)**	85.89 ± 0.70	70.30 ± 0.90	p < 0.001
**Fat mass (kg)**	9.24 ± 0.71	22.52 ± 1.15	p < 0.001
**Fat percentage (%)**	14.11 ± 0.70	29.70 ± 0.90	p < 0.001
**Body cell mass (kg)**	30.77 ± 0.45	26.58 ± 0.38	p < 0.001
**Extracellular mass (kg)**	22.69 ± 0.50	21.72 ± 0.33	-
**Protein mass (kg)**	9.24 ± 0.33	9.07 ± 0.14	-
**Mineral mass (kg)**	3.24 ± 0.12	3.71 ± 0.06	p < 0.001
**Muscle mass (kg)**	27.24 ± 0.48	21.65 ± 0.30	p < 0.001
**Total body Potassium (g)**	146.89 ± 2.13	117.52 ± 1.68	p < 0.001
**Total body Calcium (g)**	1185.32 ± 15.42	972.87 ± 12.11	p < 0.001
**Glycogen (g)**	521.64 ± 7.55	450.36 ± 6.38	p < 0.001

Values represent mean ± SEM. All male subjects were compared to female subjects according to their BIA characteristics. Student t-tests were used and p < 0.05 considered as statistically significant.

### Combined HIV-positive and HIV HAART participant and body composition characteristics

To gain further insight into body composition changes, we next compared the HIV-positive HAART versus HAART-naïve groups but independently of gender. The HIV-positive HAART group displayed a greater W:H ratio suggesting some degree of metabolic remodeling with treatment (Table 3). Although both WC and HC were lower in the HAART group, the percentage decrease was higher for the HC thereby explaining the higher W:H ratio found. The BIA analysis demonstrates that protein and mineral content levels were reduced with HAART (p < 0.05 for all) (see Table 4).

**Table 3:** **Summary of anthropometric characteristics for all participants in the HIV-positive and HIV-positive HAART treated groups, irrespective of gender**

**Variable**	**HIV-positive**	**HIV-HAART**	***p*** **-value**
	**(n = 48)**	**(n = 98)**	
**Age (years)**	30.15 ± 0.86	34.38 ± 0.53	p < 0.05
**Height (cm)**	162.00 ± 1.10	171.62 ± 0.90	-
**Weight (kg)**	71.68 ± 2.25	60.00 ± 1.70	-
**BMI (kg/m** ^**2**^ **)**	27.38 ± 0.94	20.42 ± 0.70	-
**Triceps skinfold (mm)**	24.44 ± 2.08	7.03 ± 1.42	-
**Mid-upper arm circumference (cm)**	30.30 ± 0.80	25.90 ± 0.58	-
**Waist circumference (cm)**	87.41 ± 2.13	76.30 ± 1.60	-
**Hip circumference (cm)**	103.84 ± 1.97	87.10 ± 1.50	p < 0.05
**Waist-to-height ratio**	0.54 ± 0.01	0.45 ± 0.01	-
**Waist-to-hip ratio**	0.84 ± 0.01	0.88 ± 0.009	p < 0.05

Values represent mean ± SEM. HIV-positive and HIV-HAART subjects were compared and Student t-tests were used and p < 0.05 considered as statistically significant.

**Table 4:** **Summary of BIA characteristics for all participants in the HIV-positive and HIV-positive HAART treated groups, irrespective of gender**

**Variable**	**HIV-positive**	**HIV-HAART**	***p*** **-value**
	**(n = 48)**	**(n = 98)**	
**CD4**	323.00 ± 34.10	426.30 ± 25.20	-
**Body cell mass: Fat free mass**	0.56 ± 0.002	0.58 ± 0.002	-
**Resting metabolic rate (kcal)**	1647.00 ± 23.00	1742.80 ± 15.30	-
**Fat free mass (kg)**	51.20 ± 1.04	51.89 ± 0.73	-
**Fat free mass (%)**	73.20 ± 1.50	86.88 ± 1.09	-
**Fat mass (kg)**	20.40 ± 1.67	8.16 ± 1.19	-
**Fat percentage (%)**	26.80 ± 1.52	13.12 ± 1.09	-
**Body cell mass (kg)**	28.47 ± 0.58	29.90 ± 0.41	-
**Extracellular mass (kg)**	22.72 ± 0.48	21.90 ± 0.34	-
**Protein mass (kg)**	9.60 ± 0.21	8.71 ± 0.18	p < 0.05
**Mineral mass (kg)**	3.77 ± 0.084	3.05 ± 0.07	p < 0.05
**Muscle mass (kg)**	23.90 ± 0.58	26.35 ± 0.40	-
**Total body Potassium (g)**	129.00 ± 3.10	142.90 ± 2.11	-
**Total body Calcium (g)**	1055.80 ± 22.20	1156.70 ± 15.25	-
**Glycogen (g)**	482.60 ± 9.85	507.54 ± 6.92	-

Values represent mean ± SEM. HIV-positive and HIV-HAART groups were compared. Student t-tests were employed and p < 0.05 considered as statistically significant.

### Female specific participant and body composition characteristics

When the study groups were further sub-divided to take into account the duration of treatment, we established that female participants on HAART for less than 3 years exhibited a decrease in several body composition parameters (Table 5). In agreement most of the BIA characteristics were significantly attenuated in the HAART group (Table 6). For male participants, no significant differences were found between the HAART-naïve and HAART groups (data not shown). Together these data suggest some degree of muscle wasting in females receiving HAART during the first three years of treatment.

**Table 5:** **Summary of anthropometric characteristics for females in the HIV-positive and HIV-HAART (<3 years) group**

**Variable**	**HIV-positive**	**<3 years**	***p*** **-value**
	**(n = 35)**	**(n = 29)**	
**Age (years)**	29.40 ± 1.05	29.66 ± 1.14	-
**Height (cm)**	159.06 ± 1.04	156.24 ± 1.32	-
**Weight (kg)**	73.90 ± 2.79	59.94 ± 2.87	p < 0.001
**BMI (kg/m** ^**2**^ **)**	29.15 ± 1.10	24.43 ± 1.08	p < 0.01
**Triceps skinfold (mm)**	29.30 ± 2.19	20.63 ± 1.83	p < 0.01
**Mid-upper arm circumference (cm)**	31.30 ± 1.00	27.68 ± 1.08	p < 0.05
**Waist circumference (cm)**	91.30 ± 2.46	83.46 ± 2.60	p < 0.05
**Hip circumference (cm)**	108.50 ± 2.07	97.71 ± 2.49	p < 0.01
**Waist-to-height ratio**	0.57 ± 0.02	0.53 ± 0.02	-
**Waist-to-hip ratio**	0.84 ± 0.01	0.85 ± 0.01	-

Values represent mean ± SEM. Student t-tests were employed and p < 0.05 were considered as statistically significant.

**Table 6:** **Summary of BIA characteristics for females in the HIV-positive and HIV-HAART (<3 year) group**

**Variable**	**HIV-positive**	**<3 years**	***p*** **-value**
	**(n = 35)**	**(n = 29)**	
**CD4**	348.62 ± 41.89	307.89 ± 32.06	-
**Body cell mass: Fat free mass**	0.55 ± 0.001	0.55 ± 0.002	-
**Resting metabolic rate (kcal)**	1579.26 ± 17.00	1498.41 ± 20.05	p < 0.01
**Fat free mass (kg)**	49.40 ± 1.08	44.16 ± 1.21	p < 0.01
**Fat free mass (%)**	68.52 ± 1.35	75.86 ± 1.79	p < 0.01
**Fat mass (kg)**	24.40 ± 1.83	15.83 ± 1.79	p < 0.01
**Fat percentage (%)**	31.49 ± 1.35	24.15 ± 1.79	p < 0.01
**Body cell mass (kg)**	27.16 ± 0.58	24.39 ± 0.63	p < 0.01
**Extracellular mass (kg)**	22.24 ± 0.50	19.78 ± 0.59	p < 0.01
**Protein mass (kg)**	9.52 ± 0.20	8.24 ± 0.29	p < 0.001
**Mineral mass (kg)**	3.89 ± 0.008	3.38 ± 0.12	p < 0.001
**Muscle mass (kg)**	22.23 ± 0.46	19.82 ± 0.54	p < 0.01
**Total body Potassium (g)**	120.10 ± 2.60	107.77 ± 2.81	p < 0.01
**Total body Calcium (g)**	991.54 ± 18.77	902.38 ± 20.31	p < 0.01
**Glycogen (g)**	460.34 ± 9.89	413.24 ± 10.71	p < 0.01

Values represent mean ± SEM. Student t-tests were employed and p < 0.05 were considered as statistically significant.

### Triceps skinfolds and W:H ratio

**Figure 1:**
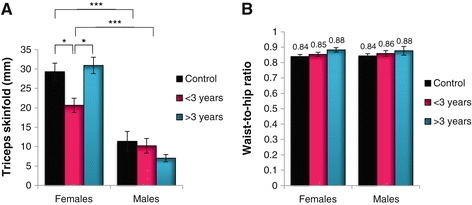
Anthropometric analysis investigating triceps skinfold (A) and waist-to-hip ratio (B) according to gender and treatment (*p < 0.05; **p < 0.01; ***p < 0.001).

To further understand the nature of body composition changes found in female participants, we next compared anthropometric and BIA data in a temporal fashion (HAART <3 years versus >3 years) for both genders. Here factorial analysis of variance (ANOVA) indicated a main effect of gender (p < 0.001), and treatment and gender (p < 0.05). Overall, females displayed significantly larger TSFs compared to males irrespective of treatment status (Figure 1A). Thus, even though the female TSF values decreased during the earlier period of HAART, this was still significantly higher than their male counterparts at the same stage. Unlike male participants, TSF values with longer treatment duration for females were similar to that of the HAART-naïve group. However, this trend was not present with the W:H ratio data, where no significant differences were observed between any of the groups (Figure 1B).

### Body cell mass (BCM), fat free mass (FFM), protein mass (PM), fat mass (FM) and muscle mass (MM)

**Figure 2:**
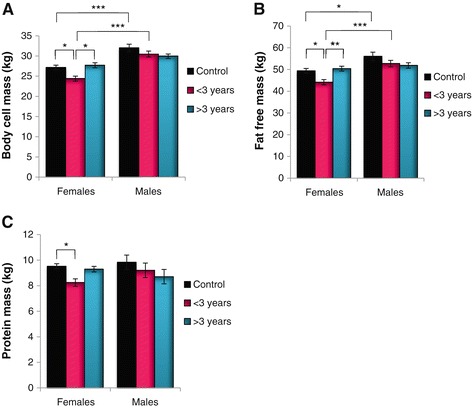
**BIA characteristics according to gender and treatment. (A)** Body cell mass; **(B)** Fat free mass; and **(C)** Protein mass (*p < 0.05; **p < 0.01; ***p < 0.001).

The BIA analyses showed analogous patterns for the female participants, i.e. lower BCM early on followed by recovery with longer HAART (Figure 2A). A main effect of treatment (p < 0.05), gender (p < 0.001), and treatment and gender (p < 0.05) was evident when BCM was analyzed using factorial ANOVA. Bonferroni *post-hoc* tests indicated significant differences in the female treatment groups, however, males displayed a higher BCM overall, except for the >3 year group. The FFM data demonstrated a similar arrangement, with effects of treatment (p < 0.05), gender (p < 0.001) and treatment and gender (p < 0.05) (Figure 2B).

**Figure 3:**
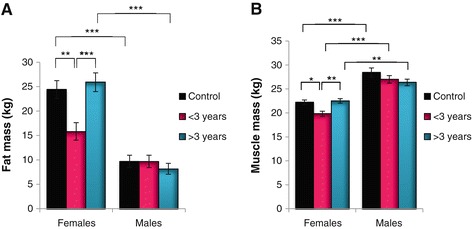
Fat mass (A) and Muscle mass (B) according to gender and treatment (*p < 0.05; **p < 0.01; ***p < 0.001).

Similar findings were established for PM, and a main effect of treatment (p < 0.05) was evident according to factorial ANOVA (Figure 2C). In line with our findings for the other parameters studied here, PM for females in the <3 year group was attenuated versus controls (p < 0.05). Females generally displayed a higher FM compared to their male counterparts (Figure 3A) and the trend was like before. However, the male participants started with a markedly reduced FM that remained relatively unchanged for the duration of HAART. Factorial ANOVA indicated a main effect of gender (p < 0.001), and treatment and gender (p < 0.05). Here gender differences were evident between the controls and the >3 year HAART treatment groups (p < 0.001 for both). For MM, analogous patterns were found for female participants as before, although males generally exhibited a greater MM compared to females (Figure 3B). A main effect of treatment (p < 0.05), gender (p < 0.001) and treatment and gender (p < 0.05) was evident.

### W:H ratio and CD4 cell count

**Figure 4:**
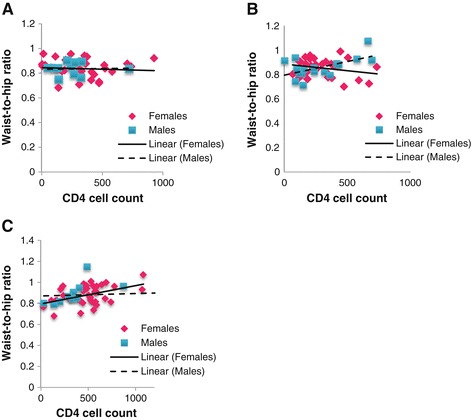
**Correlation between males and females for the HIV-positive group (A), the <3 year HAART treatment group (B) and >3 year HAART treatment group (C) with regards to Waist-to-hip ratio and CD4 cell count.**

We also performed correlations for W:H ratio versus CD4 count (between genders) for the HAART-naïve and HAART (<3 years, >3 years) groups, respectively. The HAART-naïve group did not reveal any significant correlation between W:H ratio and CD4 count for males or females (Figure 4A). Unlike the females, a significant moderate positive correlation was found for males (r = 0.56; p < 0.05) for the early HAART group (<3 years) (Figure 4B). However, with longer duration of HAART (>3 years) this effect was abolished in the male participants while females exhibited a moderate positive correlation (r = 0.46; p < 0.05) (Figure 4C).

## Discussion

This study found striking gender-based anthropometric and BIA characteristic differences in black South African HIV-positive individuals on HAART. The data reveal that females are mostly overweight by the time HAART is initiated, followed by a period of muscle wasting, whereafter (>3 years HAART) weight levels return to baseline levels. Although not significant overall, the females (compared to the males but also within the female treatment groups) displayed elevated W:H ratios after prolonged HAART, therefore potentially putting them at increased risk for the future onset of cardio-metabolic complications.

Our finding that the majority of black HIV-positive women (at start of HAART) displayed obesity is in broad agreement with others [16] who reported that the prevalence of obesity is sharply increasing within the larger South African population. Here they found a greater prevalence of obesity in females compared to males (56% versus 29%) (according to BMI cut-off values), higher than what is typically observed for other African countries [12,16]. Since some African cultures encourage overeating and associate the consumption of luxury foodstuffs (usually high fat content) with social status [17], cultural and lifestyle choices may help explain higher obesity levels in HIV-infected females here studied. In addition, black women are less pressured to be slim and relate thinness with illness and HIV-AIDS [17,18].

We also found elevated TSF values in females compared to males indicating an elevated risk for obesity and related cardiovascular risk. Moreover, when the total group was sub-divided into HAART-naive, <3- and >3 years HAART distinct differences were observed only in the female treatment categories. These findings are in agreement with others who found significantly lowered TSF values in Indian female HIV-positive patients following 12 months of HAART [19]. Likewise George *et al.* [19] reported decreased TSF values in a black South African HIV-positive population (18–55 years, 65% female) until 27 months after anti-retroviral treatment initiation [20]. For the current study, females also exhibited a significant degree of muscle wasting at the <3 years HAART time point. A similar biphasic pattern was observed for BCM, FFM, and FM. Why the initial reduction in such parameters following HAART? Some suggested that endocrine, environmental and socio-economic factors may be potential explanations [21]. However, we propose that the decrease in the anthropometric and BIA characteristics within the first three years of treatment can most likely be attributed to the stabilization of the immune system and metabolism that occur as a result of the infection and the introduction of HAART. Moreover, oxidation of visceral fat for energy utilization could be an additional factor to help explain the reduction in waist and hip circumferences (<3 years HAART). Furthermore, with any chronic disease (especially HIV infection) the resting energy expenditure (REE) is enhanced [22]. Such findings are of clinical significance since an elevated REE may also impair muscle mass gain [23]. However, further studies are required to ascertain whether these proposals are valid and to also evaluate nutritional status/food security in this particular population.

Literature suggests that both TSF and mid-upper arm circumference (MUAC) are independent predictors for survival, and surrogate parameters for muscle wasting [24,25]. Both parameters can also be employed to determine the risk for possible co-infections [25]. For males we observed no differences for TSF or MUAC as treatment duration increased. Moreover, the initial attenuation in TSF and MUAC (<3 years HAART) for females returned to baseline levels suggesting that long-term HAART may lower the risk for co-infections and thus increase survival rates.

Muscle mass, FFM and FM did not significantly differ in males for the different HAART groups compared to the HAART-naïve one. Although there is a tendency for weight loss amongst males in the <3 year HAART compared to the HAART-naïve group (p = 0.08) (data not shown), the variability in weight in the >3 year HAART group makes comparisons non-significant. However, inclusion of more subjects (particularly males) may shed greater light on this question.

Waist-to-hip ratios were also assessed as a marker for cardio-metabolic risk [26,27]. Females in the HAART-naïve and treatment groups presented with a higher risk for cardio-metabolic complications since W:H ratios were above standard cut-off levels [28,29]. However, males were not at risk since the W:H ratios were lower than standard cut-off criteria [29]. Since females appear to be at greater risk, we also determined the potential relationship between central obesity and CD4 T-cell counts. The proposed association between central obesity (W:H ratio) and CD4 can be explained by leptin (secreted by adipocytes) aiding in the proliferation of CD4+ T-cells [30]. Here the weak correlation for females in the <3 year HAART group was expected since most of the parameters investigated were decreased at this time point. However, with prolonged HAART females displayed a higher risk for future cardio-metabolic dysfunction.

We acknowledge that the present study has some limitations, one being the intrinsic limitation of a cross-sectional design. Although our findings demonstrated clear gender-based differences, another limitation is the relatively low number of male participants (compared to females). This observation is in accordance with previous work where males were also less likely to attend clinics and participate in research studies [31]. This is mainly due to males being the sole breadwinner and thus unable to take time off from work to receive medical care and/or participate in research projects. Although nutritional status would have provided valuable insight into caloric intake and further strengthened our results, this could not be performed due to logistical reasons and lengthy patient contact time required. Finally, this study lacked an HIV-negative control group to compare an HIV-infected population with a non-infected one.

## Conclusions

The current study established unique gender-based differences in a peri-urban black South African HIV-infected population. Unlike males, females displayed initial muscle wasting but thereafter gained weight (significantly higher W:H ratios) with prolonged treatment. Thus these data suggest that female HIV-positive individuals on longer-term HAART may be at greater risk for the development of cardio-metabolic disease complications. Future studies should focus on determining the cause of the body compositional changes undergone with treatment duration and distinguish between the influence of HIV itself and the treatment thereof. The multiple anthropometric and body compositional characteristics described provided robust insights into changes observed with increasing HAART duration. This reinforces the importance of basic anthropometric measurements that are fairly quick, cost-effective and easy to perform in peri-urban and rural settings to aid in identifying patients at risk for HAART-related co-morbidities.

## Abbreviations

HIV: Human immunodeficiency
AIDS: Acquired immune deficiency syndrome
HAART: highly active antiretroviral therapy
>: More than
<: Less than
W:H: Waist to hip ratio
CVD: Cardiovascular disease
TSF: Triceps skinfold
ANOVA: Analysis of variance
BIA: Bioelectrical impedance analysis
BCM: Body cell mass
PM: Protein mass
FM: Fat mass
CD4: Cluster of differentiation 4
FFM: Fat free mass
REE: Resting energy expenditure
MUAC: Mid-upper arm circumference
